# Biosynthesis and analytics of specialized pro-resolving mediators: facts and faults

**DOI:** 10.1016/j.jlr.2026.101133

**Published:** 2026-09-11

**Authors:** Nils Helge Schebb, Hartmut Kühn, Nadja Kampschulte, Nicolas Flamand, Marc Peters-Golden, Per-Johan Jakobsson, Hans-Erik Claesson, Karsten H. Weylandt, Nadine Rohwer, Meike J. Saul, Eugen Proschak, Bernhard Brüne, Ulrike Garscha, Thorsten J. Maier, Paola Patrignani, Miguel A. Gijón, Francisco J. Schopfer, Stacy L. Gelhaus, Michael H. Gelb, Robert C. Murphy, Carsten Skarke, Alan R. Brash, Bruce A. Freeman, Ian A. Blair, Derek W. Gilroy, Gerd Geisslinger, Julien Hanson, Kim Ekroos, Garret A. FitzGerald, Astrid S. Kahnt, Valerie B. O’Donnell, Mohamad Wessam Alnouri, Stefan Offermanns, Dieter Steinhilber

**Affiliations:** 1 Food Chemistry, Faculty of Mathematics and Natural Sciences, University of Wuppertal, Wuppertal, Germany; 2 Department of Biochemistry, Charité-Universitätsmedizin Berlin, Corporate Member of Freie Universität Berlin and Humboldt-Universität zu Berlin, Berlin, Germany; 3 Centre de recherche de l’Institut universitaire de cardiologie et de pneumologie de Québec, Département de médecine, Faculté de médecine, Université Laval, Québec City, Quebec, Canada; 4 Division of Pulmonary and Critical Care Medicine, Department of Internal Medicine, University of Michigan Medical School, Ann Arbor, MI, USA; 5 Division of Rheumatology, Department of Medicine Solna, Karolinska Institutet & Karolinska University Hospital, Center for Molecular Medicine, Stockholm, Sweden; 6 Department of Medicine, Karolinska University Hospital Solna, Stockholm, Sweden; 7 Division of Gastroenterology & Endocrinology and Nutrition Skills Lab, University Hospital Ruppin-Brandenburg, Brandenburg Medical School, Neuruppin, Germany; 8 Faculty of Health Sciences, Joint Faculty of the Brandenburg University of Technology, Brandenburg Medical School and University of Potsdam, Potsdam, Germany; 9 Department of Molecular Toxicology, German Institute of Human Nutrition Potsdam-Rehbruecke, Nuthetal, Germany; 10 Department of Oncology, Hematology and Bone Marrow Transplantation with Section Pneumology, University Medical Center Hamburg-Eppendorf, Hamburg, Germany; 11 Institute of Pharmaceutical Chemistry, Goethe University Frankfurt, Frankfurt, Germany; 12 Fraunhofer Institute for Translational Medicine and Pharmacology, ITMP and Fraunhofer Cluster of Excellence for Immune mediated diseases, CIMD, Frankfurt am Main, Germany; 13 Institute of Biochemistry I, Faculty of Medicine, Goethe University Frankfurt, Frankfurt, Germany; 14 Department of Pharmaceutical/Medicinal Chemistry, Institute of Pharmacy, Greifswald University, Greifswald, Germany; 15 Department of Immunology, Paul-Ehrlich-Institute (Federal Institute for Vaccines and Biomedicines), Langen, Germany; 16 Department of Neuroscience, Imaging and Clinical Sciences, and Center for Advanced Studies and Technology (CAST), “G. d’Annunzio” University, Chieti, Italy; 17 Cayman Chemical Company, Ann Arbor, MI, USA; 18 Department of Pharmacology and Chemical Biology, University of Pittsburgh, School of Medicine, Pittsburgh, PA, USA; 19 Department of Pharmacology and Chemical Biology, Health Sciences Mass Spectrometry Core, University of Pittsburgh, Pittsburgh, PA, USA; 20 Department of Chemistry and Biochemistry, University of Washington, Seattle, WA, USA; 21 Department of Pharmacology, University of Colorado-Denver, Aurora, CO, USA; 22 Institute for Translational Medicine and Therapeutics, Perelman School of Medicine, University of Pennsylvania, Philadelphia, PA, USA; 23 Department of Pharmacology, Vanderbilt University, Nashville, TN, USA; 24 Penn/CHOP Friedreich Ataxia Center of Excellence, Perelman School of Medicine, University of Pennsylvania, Philadelphia, PA, USA; 25 Department of Systems Pharmacology and Translational Therapeutics, Perelman School of Medicine, University of Pennsylvania, Philadelphia, PA, USA; 26 Dept. of Ageing, Rheumatology and Regenerative Medicine, University College London, London, UK; 27 Institute of Clinical Pharmacology, Faculty of Medicine, Goethe-University Frankfurt, Frankfurt am Main, Germany; 28 Laboratory of Molecular Pharmacology, GIGA Research Institute & CIRM, University of Liège, Liège, Belgium; 29 Lipidomics Consulting Ltd., Esbo, Finland; 30 Systems Immunity Research Institute, School of Medicine, Cardiff University, Cardiff, UK; 31 Max Planck Institute for Heart and Lung Research, Department of Pharmacology, Bad Nauheim, Germany; 32 Center for Molecular Medicine, Goethe University Frankfurt, Frankfurt, Germany

**Keywords:** lipoxygenase, leukotriene, resolving, lipoxin, SPM receptor, resolution

## Abstract

Specialized pro-resolving mediators (SPMs) have been suggested to act as key factors that mediate the resolution of inflammation. With the availability of synthetic standards, pharmacological studies have garnered support for the hypothesis. However, assessment of the catalytic properties of lipoxygenases as well as investigations of the signaling of the proposed SPM receptors raise serious doubt that SPMs are endogenous mediators of the resolution of inflammation. In line with this, careful evaluation of published data on SPM detection revealed a lack of evidence for many reported SPMs in biological samples due to analytical LC-MS/MS methods that do not meet the quality standards of the scientific community, or they rely on ELISAs that lack selectivity and are not suited for the establishment of the presence of SPMs in complex biological samples. Furthermore, artefacts due to autoxidation of PUFAs can be misinterpreted as SPM formation. In this review, we critically assess the published reports on the biosynthesis and analysis of SPMs and summarize the current status in the field.

Oxylipins are formed by lipoxygenase (LOX), cyclooxygenase and cytochrome P450 enzymes from polyunsaturated fatty acids (PUFA), predominantly linoleic (LA), arachidonic (AA), eicosapentaenoic (EPA), and docosahexaenoic (DHA) acids. The prostaglandins and leukotrienes are mediators involved in the regulation of various physiological functions, including pro-inflammatory roles that are countered in therapeutics by NSAIDS or receptor antagonists. With contrasting activities, a subset of oxylipins, termed specialized pro-resolving lipid mediators (SPMs) are proposed to support the resolution of inflammation (1, 2, 3, 4). SPMs such as lipoxins (LX), resolvins (Rv), maresins (MaR) and protectins (PD) are proposed to be formed by LOX from AA, EPA or DHA (Fig. 1). However, a first report that failed to detect SPMs in human plasma and urine even after supplementation of patients with EPA and DHA was published in 2015 (5). Data consistent with the null hypothesis were subsequently published from other laboratories (reviewed in (6)). Apart from patient data, there is accumulating evidence from biochemical assays and cell assays that human leukocytes lack the biosynthetic capacities to produce relevant levels of SPMs. Furthermore, analytical methods to detect SPMs in biological samples have been challenged (6, 7, 8). This review provides an update of the SPM biosynthesis and analytics highlighting the ongoing problems with SPM detection in biological samples by liquid chromatography and tandem mass spectrometry (LC-MS/MS) methods and ELISAs. Overall, it can be concluded that evidence for enzymatic formation of most SPMs from endogenous PUFA substrates in cells and tissues is missing and that autoxidation can represent a significant artefactual source of the oxylipins.

**Fig. 1:**
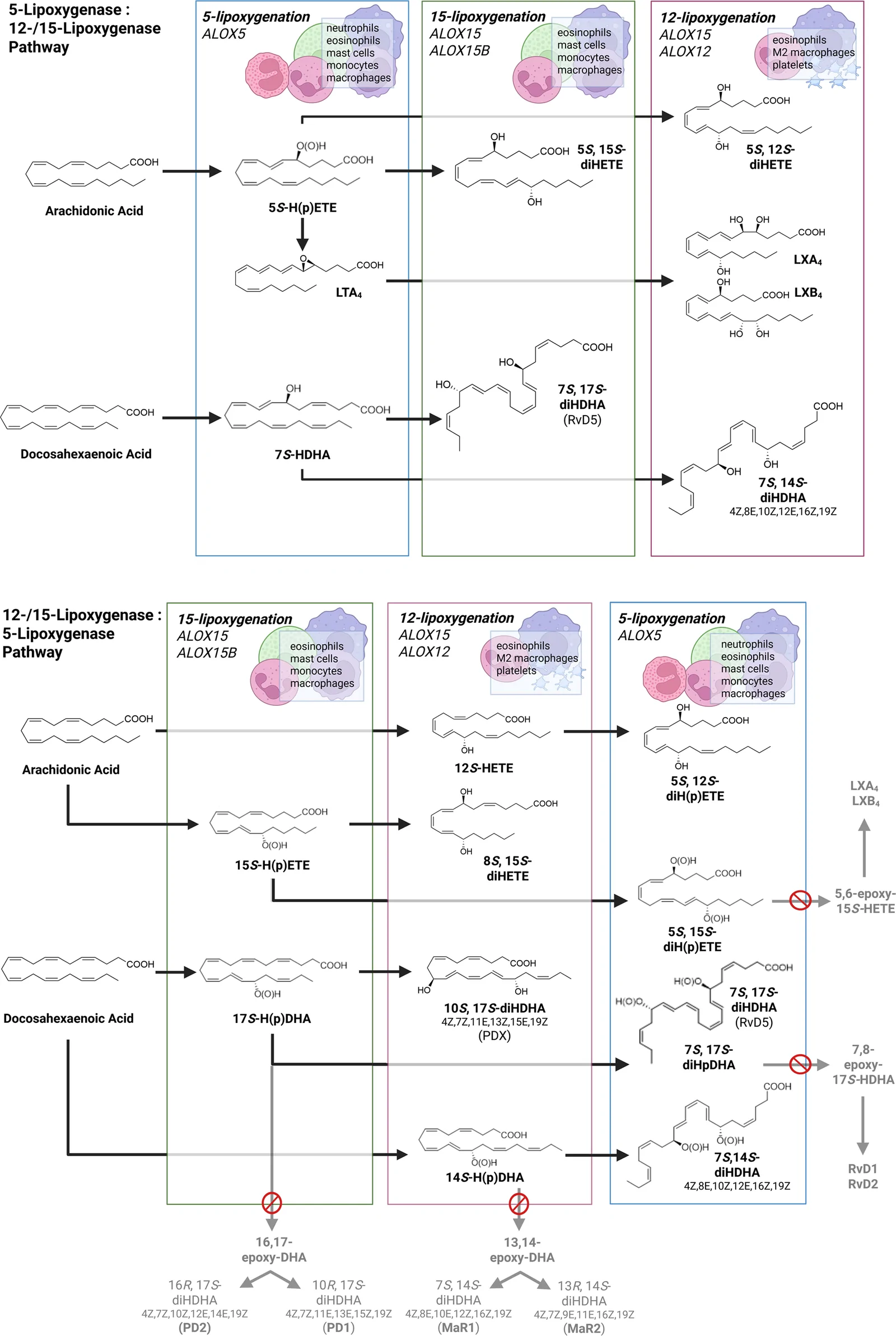
Proposed biosynthetic pathways for SPMs. The red circles indicate reactions which are not enzymatically supported. The epoxy fatty acid intermediates refer to the LOX-mediated allylic epoxides proposed to be formed from the respective fatty acid hydroperoxide precursor.

## Analytical Detection of SPMs

### Analytical detection of SPMs using LC-MS/MS, pre-2022

Up to around 2022, only a small number of analytical laboratories routinely reported the detection of SPMs in biological samples using LC-MS/MS (9, 10, 11, 12). Several other laboratories monitored for the presence of SPMs using the same oxylipin platforms, but these lipids were not conclusively found (5, 13, 14). For example, a study that showed primary data 10 years ago concluded that even with fish oil supplementation, convincing signals for SPMs were not detected in plasma or urine of healthy subjects (5). Unfortunately, negative data indicating the absence of SPMs in biological samples was not routinely published, making evaluation of the issue difficult. In 2023, it was noted in a Matters Arising that the laboratories routinely detecting SPMs using LC-MS/MS were interpreting their data differently from community accepted approaches, specifically by (i) assigning structures based on low-quality MS/MS spectra containing putative “diagnostic ions”, (ii) not requiring the presence of a chromatographic peak, and/or (iii) using a single area value (cps), generated by integrating noise, as the LOD/LOQ value for all analytes, for example, 2000 cps (7, 8, 15). It has since then been clearly shown through independent reanalysis of data using these methods that they are not consistent with good practice in the field of small molecule mass spectrometry and can routinely infer the presence of molecules in samples where evidence does not exist (7, 16, 17, 18). For example, in 2022, reanalysis of data from Dalli *et al.* on arthritis patient plasma failed to confirm the presence of SPMs (7). Following this, raw data from a second study originally claiming to detect SPMs in human skin blisters (19) were independently reassessed. The new analysis demonstrated that no SPM signals had been present in the original raw data (17, 20). Finally, a recent reanalysis of a third study by a different independent team concluded that the presence of SPMs in plasma from mice with inflammatory arthritis was not supported by detectable signals (18). In summary, three independent reanalyses of raw data by different groups of scientists have now raised serious concerns relating to ongoing studies on SPMs.

Further complicating the assessment of published evidence, it was noted that around 80 publications dating from around 2016 to 2020 contained figures of what were labeled as chromatographic traces of tissue SPMs. These were subsequently clarified by the authors to represent “illustrations” or “schematics”, being shown in place of actual data (21, 22). Furthermore, as reported in an editorial note in 2025, around 15 of these included the same illustration, labelled as different tissues across the studies (20). A small number have been corrected by clarifying that illustrated peaks were not real data (23, 24, 25, 26). However, these corrections did not clarify why the underlying analytical data originally referred to in the figure legends were not shown, nor did they provide the raw data (23, 24, 25, 26). Because for the majority of these studies, the raw data remain missing, the presence of SPMs in these still cannot be established. For the few that included chromatograms in corrections/addendums, the data quality is mixed. For example, in Mai *et al.*, many peaks are of insufficient quality to support the presence of SPMs (27), while the correction to Werz *et al.* adequately shows that RvD2 and RvD5 or its isomers can be detected in human macrophage lipid extracts (28).

### Post-2022 recommendations for oxylipin analysis and their application to SPM studies

In 2024, the International Lipidomics Society established an interest group (Oxylipin Analysis IG) as a community-led effort to formulate and publish a set of recommendations on LC-MS/MS analysis of oxylipins for research applications in addition to the existing minimal reporting checklist (29). The main aim was to support researchers to set up methods and process their raw data in line with community best practice for LC-MS/MS methods, noting that these points would equally apply to analysis of all small molecules using this methodology. In this context, the focus was on quantitation conducted in research settings which cannot fully conform to clinical guidelines for reasons that include limited availability of matrix and lack of availability of analyte-free matrix for validation. A guideline co-authored by around 100 analysts was published early in 2025 (16). Summarizing this, several key principles were agreed.

(i) the need to use raw/unsmoothed data to calculate signal-to-noise ratios (S/N).
(ii) the various ways in which S/N can be calculated, with recommended values for LOD and LOQ of at least 3:1 and 5:1, respectively, based on the standard approach of H/h (H: height of peak, h: height of noise).
(iii) that all oxylipins being formally quantified should be included as primary standards in standard curves.
(iv) that secondary/tertiary MRMs should be used for identification purposes, especially for lower abundance oxylipins.

It was also noted that approaches recommended by clinical guidelines based on variance cannot be applied *only in part* to research assays, as that can lead to significant over-interpretation of S/N or peak quality.

While writing this article, we reviewed studies published since 2022, assessing available raw data where possible, in line with the community guidelines. We found that most published studies claiming to detect SPMs in biological samples do not fully meet these recommendations. For example, several apply smoothing prior to S/N calculation (30, 31, 32) and/or lack primary standards for all analytes (33, 34, 35). Also, algorithms such as the Relative Noise Concept return higher S/N values than the standard approach (7), so that when a cutoff of ≥5 is used, signals of insufficient quality are included in the analysis (30, 31, 32).

For a small number of recent studies from Dalli *et al.*, raw LC-MS/MS data have been deposited on BioStudies supporting their reanalysis (30, 36, 37, 38, 39, 40). Examination of these datasets indicates that many of the reported SPM assignments are made on the basis of noise, and not by the presence of discernible peaks in chromatograms. Examples are shown for five of these (Figs. 2 and 3). Raw LC-MS/MS chromatograms from a study investigating MCTR compounds lack any visible peaks for MCTR1, MCTR2 and MCTR3 in macrophages, while data from another lack peaks for 6 of the 7 SPMs claimed to be detected in mouse plasma (Fig. 2) (36, 39, 41). Data in a third paper lack chromatographic peaks for many oxylipins, including SPMs from cultured cells (Fig. 2) (42). Readers are referred to PubPeer threads, which give further detail on these studies (https://pubpeer.com/publications/B56501B8F18E5C9CBD077B8FB87281; https://pubpeer.com/publications/A2869455D5251F6B32C13A20F14BE3; https://pubpeer.com/publications/095CD2B6565BB072539BD288F53063). Two additional publications from the Dalli group were assessed during drafting of this review. In one, a frameshift variant in LRG6 (an orphan receptor claimed to be specific for MaR1) was reported as associated with altered SPM generation in neutrophils (30). The most impacted lipid was claimed to be MaR2. However, the raw data on BioStudies do not evidence any detectable MaR2 in neutrophils (Fig. 3). Another study reported the detection of multiple SPMs in peritoneal lavage post vagal nerve stimulation. However, upon checking the raw data for the first SPM, RvD1, in Table 1 in (37), visible chromatographic peaks were not observed (Fig. 3), despite detectable levels being reported for the lipid. For these two papers, the full set of sample chromatograms for these two lipids are shown as screenshots (Figs. 4 and 5).

**Fig. 2:**
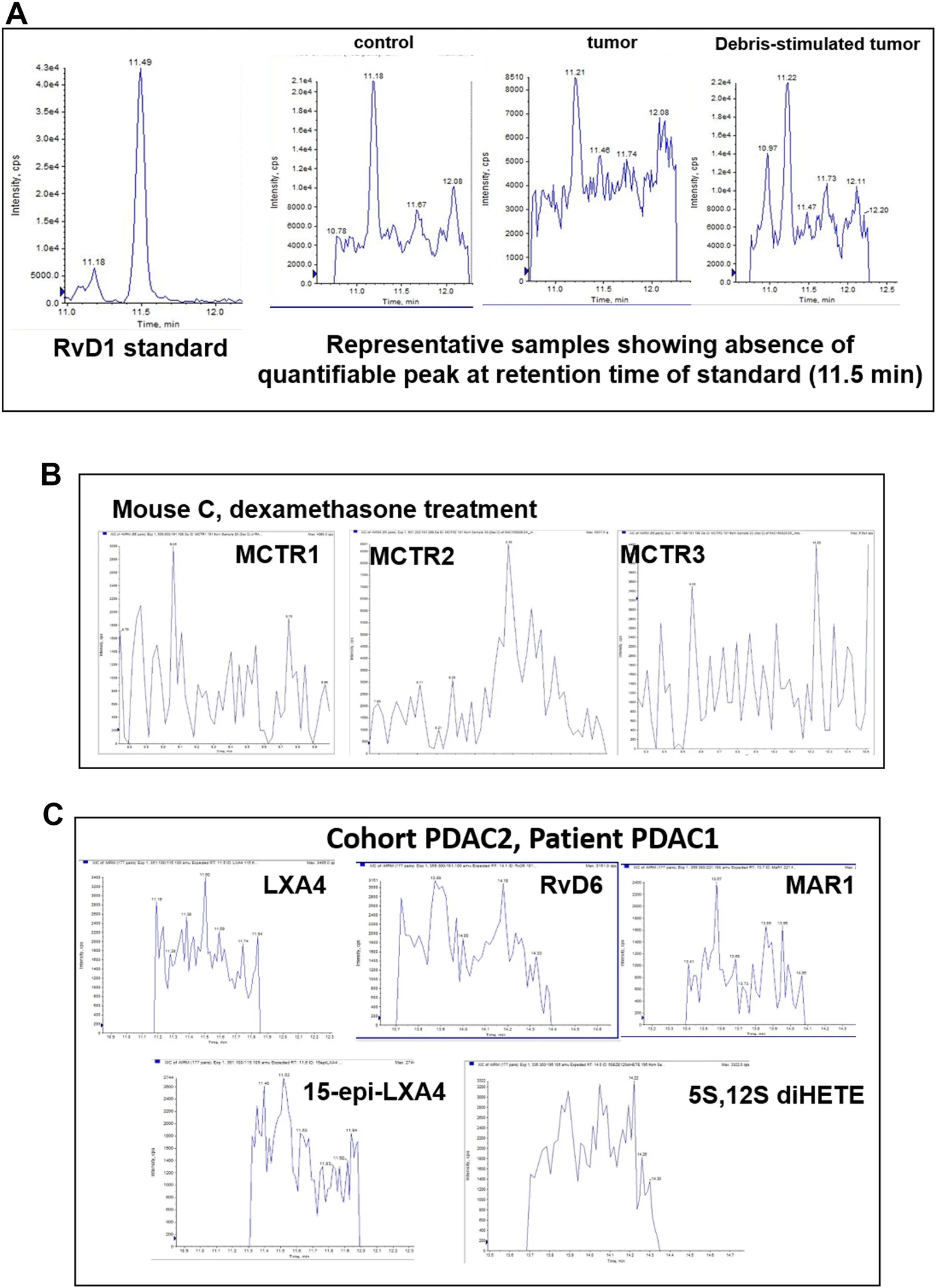
Representative data from published studies showing a lack of measurable SPMs in various tissues. Panel A. Data from Sulciner *et al.* (36) showing elution of RvD1 at 11.5 min, alongside representative data from all available samples, however none show a peak at the correct retention time for the standard. Panel B. Data from Koenis *et al.* (39). A representative mouse tissue extract is shown. No clear peaks for MCTR lipids are visible. Panel C. Data from Aguirre *et al.* (38). Chromatograms for a sample reported to contain several SPMs are shown, with no visible peaks seen.

**Fig. 3:**
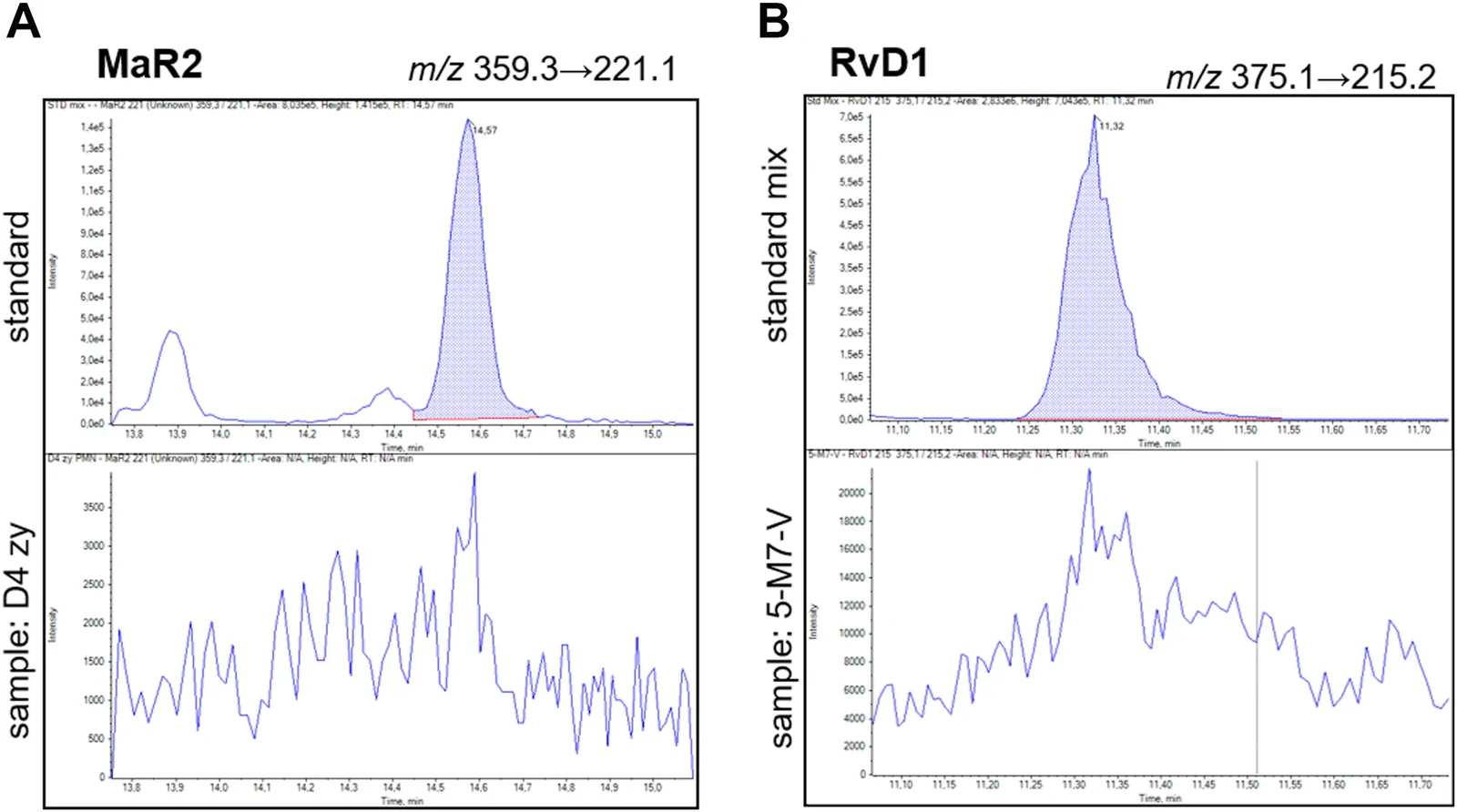
Representative data from recent studies showing lack of discernible chromatographic peaks despite the claims made of the presence of SPM in biological samples. Panel A. Signal of Maresin 2 (MaR2) MRM transition in standard and a representative sample showing no signal for this oxylipin in the supernatant of human neutrophils. Data was extracted from unsmoothed raw data provided by Gomez *et al.* (30). Panel B. Signal of resolvin D1 (RvD1) MRM transition in standard and a representative sample of peritoneal exudates of zymosan treated mice showing no signal for this oxylipin. Data was extracted from unsmoothed raw data provided by Caravaca *et al.* (37).

**Table 1:** Mono-, di- and trihydroxylated oxylipins derived from AA, EPA and DHA

Hydroxylation	Oxylipin	Fatty Acid Precursor	Hydroxy-lation	Involved or Proposed Human Enzymes
monohydroxy derivatives	5*S*-HETE^a^	AA	C5	5-LOX
	12*S*-HETE	AA	C12	12-LOX, 15-LOX1
	15*S*-HETE	AA	C15	15-LOX1, 15-LOX2
	4*S*-HDHA^b^	DHA	C4	5-LOX
	7*S*-HDHA	DHA	C7	5-LOX
	17*S*-HDHA	DHA	C17	15-LOX1, 15-LOX2
	5*S*-HEPE^c^	EPA	C5	5-LOX
	15*S*-HEPE	EPA	C15	15-LOX1, 15-LOX2
	18*R*-HEPE	EPA	C18	CYP?
dihydroxy derivatives	5,15-diHETE	AA	C5,15	15-LOX^4^:5-LOX, 5-LO:15-LOX
	8,15-diHETE	AA	C8,15	15-LOX
	resolvin D5 (RvD5)	DHA	C7,17	5-LOX:15-LOX 15-LOX:5-LOX
	resolvin D6 (RvD6)	DHA	C4,17	5-LOX:15-LOX 15-LOX:5-LOX
	maresin 1 (MaR1)	DHA	C7,14	12-LOX, 15-LOX1
	maresin 2 (MaR2)	DHA	C13,14	12-LOX, 15-LOX1
	protectin D1 (PD1)	DHA	C10,17	12-LOX, 15-LOX
	protectin D2 (PD2)	DHA	C16,17	12-LOX, 15-LOX
	protectin DX (PDX)	DHA	C10,17	12-LOX, 15-LOX
	resolvin E2 (RvE2)	EPA	C5,18	CYP?:5-LOX
	resolvin E3 (RvE3)	EPA	C17,18	CYP?
	resolvin E4 (RvE4)	EPA	C5,15	15-LOX:5-LOX, 5-LOX:15-LOX
trihydroxy derivatives	lipoxin A_4_ (LXA_4_)	AA	C5,6,15	5-LOX:12- or 15-LOX
	lipoxin B_4_ (LXB_4_)	AA	C5,14,15	5-LOX:12- or 15-LOX
	resolvin D1 (RvD1)	DHA	C7,8,17	15-LOX:5-LOX
	resolvin D2 (RvD2)	DHA	C7,16,17	15-LOX:5-LOX
	resolvin D3 (RvD3)	DHA	C4,11,17	15-LOX:5-LOX
	resolvin D4 (RvD4)	DHA	C4,5,17	15-LOX:5-LOX
	resolvin E1 (RvE1)	EPA	C5,12,18	CYP? or (15-LOX):5-LOX

a hydroxyeicosatetraenoic acid.

b hydroxydocosahexaenoic acid.

c hydroxyeicosapentaenoic acid.

**Fig. 4:**
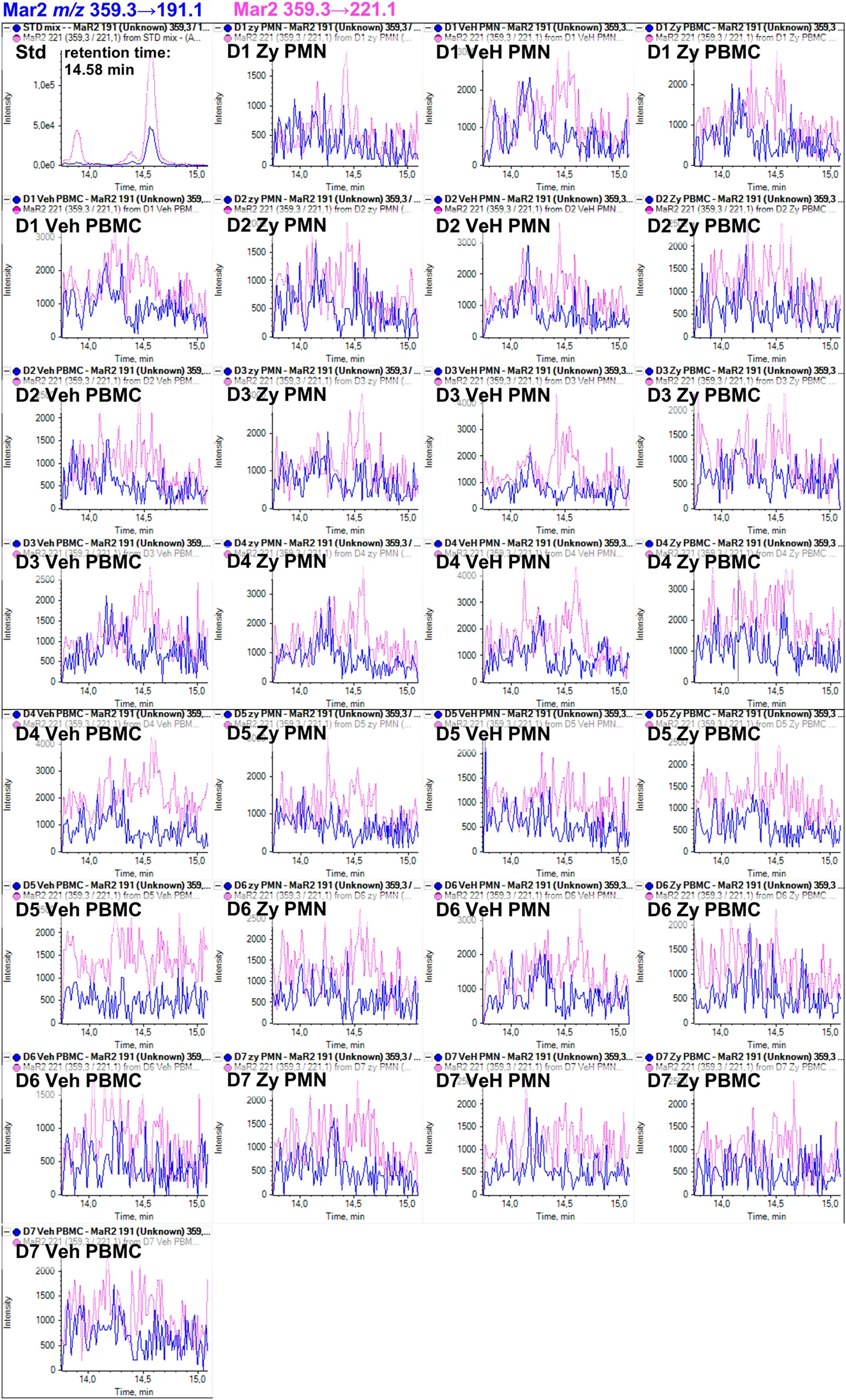
Signals of maresin 2 (MaR2) MRM transitions in standard and samples. The standard‘s retention time is 14.58 min. Data extracted from the raw data provided by Gomez *et al.* (30). Unsmoothed signals.

**Fig. 5:**
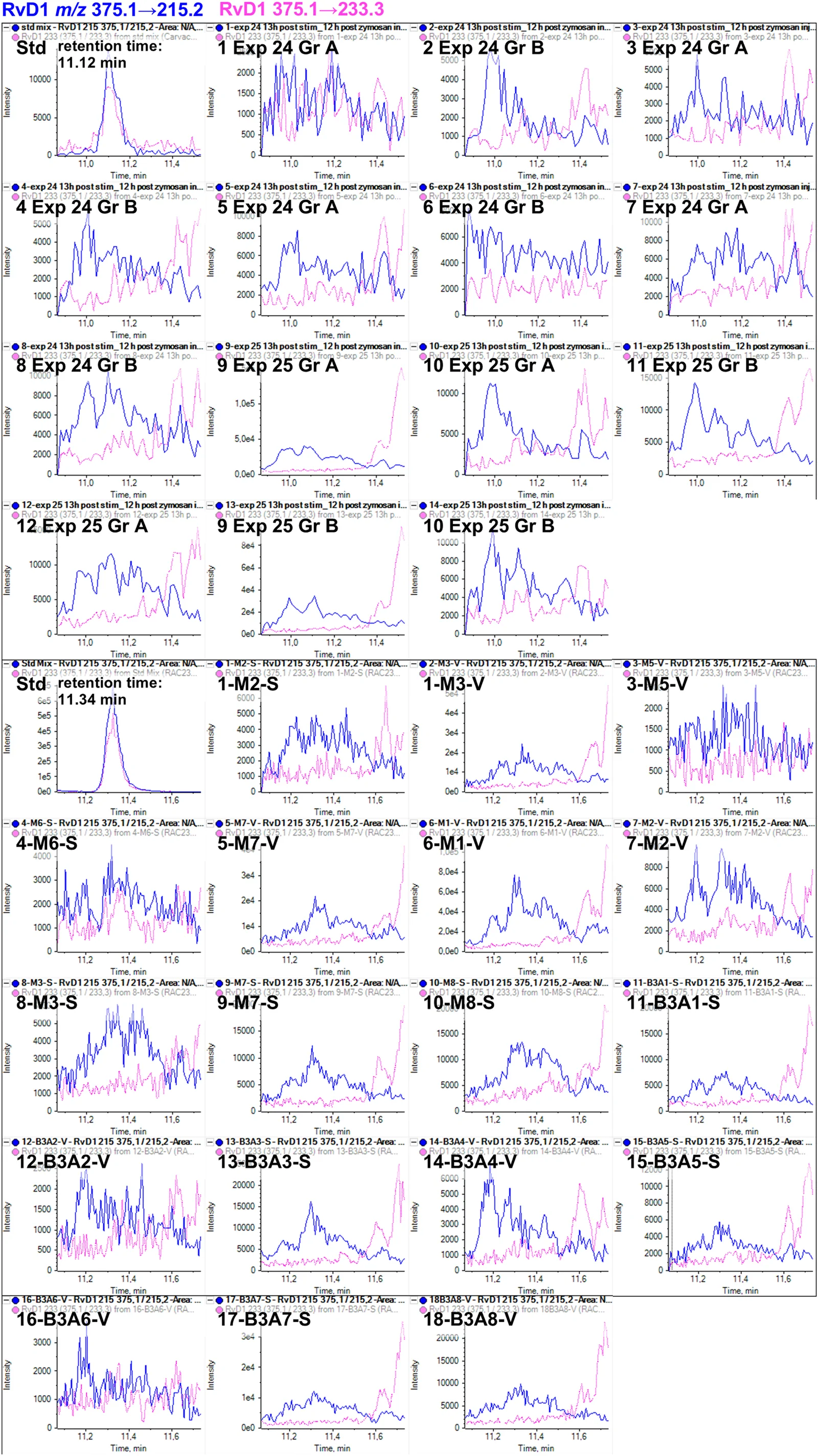
Signals of resolvin D1 (RvD1) MRM transitions in standard and samples. The standard‘s retention times are 11.12 min (sample set 1) and 11.34 min (sample set 2). Data extracted from the raw data provided by Caravaca *et al.* (37). Unsmoothed signals.

Some recent studies from Serhan, Levy and colleagues have included visible chromatographic peaks as representative data. These include publications showing endogenous D-series resolvins in human lung grafts, or lipoxins/RvE4 in dorsal air pouches in response to exogenous supplementation with high amounts of oxygenated PUFA precursors (epoxy-PUFA) (43, 44). However, while one study shows chromatographic peaks for resolvins (Fig. 1 in (43)), the MS/MS fragmentation spectra do not support structural interpretation, and no standards are shown to verify retention time. Another study shows spectra that give good visible matches with lipoxins and RvE4 (44). However, chiral analysis of RvE4 to support assignment of the lipid as the 5*S*,15*S*-diHEPE enantiomer was not performed. Neither study reports the LOD/LOQ criteria employed, precluding independent assessment of whether only chromatographic peaks of sufficient analytical quality were integrated. Last, a recent study claimed to detect 17*R*-RvD2 in human saliva and both M2 macrophages and monocytes. Closer examination of the data indicates that it is noted that (i) spectra for 17*R*-RvD2 do not match well with the synthetic standard. In particular, the large ions seen in the standard at *m/z* 175 and 215 are only weakly detected among several more intense ions that are absent in the standard, for either saliva or cell samples (Figs. 2 and 4 in (45)). These spectra do not establish the identity of the lipid. (ii) RvD2 peaks are also seen in cell extract chromatograms, with peaks of similar height to 17*R*-RvD2, and also several other isomers are present with even higher peaks in this channel (Fig. 4 in (45)). The occurrence of multiple isomers together with complex fragmentation spectra that do not closely align with the corresponding standards is more consistent with non-enzymatic lipid autoxidation than with selective enzymatic formation. Recent studies from Serhan *et al.* refer to SPM identification based on retention time and MS/MS spectral matching alone. They do not describe any criteria for assigning LOD or LOQ that are aligned with the community recommended guidelines (46). This raises questions about ongoing studies on SPMs using these methods, in particular relating to quantitation in complex tissue samples.

Some recent studies have reported RvD5, RvE4, PDX, PD1, PDX, MaR1 and LXA_4_ or its isomers in primary human macrophages under various conditions (47, 48, 49). However, these papers do not include sufficient method information and/or raw data to evidence these claims. The formation of the dihydroxy-PUFAs RvD5, RvE4 and PDX by macrophages has been confirmed by other independent labs, including through the use of chiral analysis (50, 51, 52). However, the enzymatic formation of PD1 has been fundamentally challenged (see below), with recent chiral analysis showing that PD1 is not formed by human macrophages (50, 51). Instead, co-eluting isomers generate peaks which can be misinterpreted as PD1, giving a potential explanation for why this unlikely oxylipin has been reported several times. Similarly, incorrect annotation of an isobaric peak that elutes close to MaR1 (51) could explain why this lipid has been reported in several studies. For LXA_4_, a recent study shows clear peaks using a well-documented method (52); however, its formation was not confirmed by chiral chromatography, which is particularly important for this oxylipin (see below).

In summary, available datasets provided through journal corrections and recent data depositions do not provide analytical evidence supporting the reported detection of several SPMs, beyond formation of a small number of dihydroxy-PUFA in isolated macrophages only.

### Potential for interference from SPM isomers formed by autoxidation

During the last 15 years, many studies reported oxylipin levels in plasma, including in healthy subjects, claiming either detectable levels of SPMs, or alternatively reporting extremely poor peaks that could not be integrated (5, 11, 13, 53, 54, 55, 56, 57, 58, 59, 60, 61). Aside from methodological differences outlined above, a second potential explanation for this discrepancy relates to processing and storage oxidation artefacts. Indeed, it was recently reported in two studies that fresh plasma does not contain detectable SPMs, unless incorrectly processed (50, 62). Here, chiral and reverse-phase LC-MS/MS demonstrated that the detected SPM isomers, putative RvD5, MaR1 and PDX, originated entirely from poor sample handling (50, 62).

SPMs are formally named based on their fully annotated stereochemical structures, for example, RvD1 represents the 7*S*,8*R*,17*S*-form of tri-HDHA with the double bond configuration *trans, trans,cis,trans* between the hydroxylation sites (one specific trihydroxy DHA isomer). In addition, the SPM name infers an enzymatic route to their origin by lipoxygenases (LOXs), cyclooxygenases (COXs) and cytochrome P450 enzymes (CYPs), as outlined in recent reviews and shown in Table 1 (63, 64, 65). However, taking the example of RvD1, with DHA as the substrate, there are at least 8 potential isomers based on *S/R* configuration at hydroxylation sites, and several will co-elute using reversed phase chromatography. This does not account for geometric (*cis/trans*) isomer composition which increases the number of isomers possible, and in the case of tri-hydroxy SPMs can be strongly influenced by the biosynthetic mechanism (66, 67, 68). For example, PD1 and its often more abundant isomer, PDX, differ in the configuration of the double bonds as well as chirality.

Reversed phase LC-MS/MS can determine functional group positions, but not double bond geometry or chirality. Recognizing this issue, in Technical Recommendations for Analyzing Oxylipins by Liquid Chromatography–Mass Spectrometry (16), we proposed reporting only structural information that is directly supported by analytical data. For example, using 5,15-diHEPE instead of RvE4 when the complete stereochemical structure has not been demonstrated using chiral LC-MS/MS. This is particularly an issue for complex tissues where multiple enzymes/cells are present, and autoxidation is a significant potential source of artefacts. Indeed, where multiple isomers are found, autoxidation should always be considered as a formation route, as recently shown in plasma (50, 62). Although not widely used in the oxylipin field, some groups have applied untargeted LC/MS approaches to the analysis of SPMs (69). Here, the shorthand nomenclature incorporated into LIPID MAPS should be applied, e.g. FA 20:4;O in place of 12-HETE (https://www.lipidmaps.org/databases/lmsd/LMFA03060007). This shorthand describes fatty acyl chain length, number of rings/double bonds and number of oxygenations, but not functional group, position, stereochemistry or double bond/ring configurations (70).

Raising concerns of autoxidation being a primary source of SPMs more broadly, many studies report multiple mixed isomers of SPMs in cells and tissues detected simultaneously using reverse-phase chromatography. In one, three isomers of PD1 (PD1, 17-epi-PD1 and 10*S*,17*S*-PD1) and multiple isomers of MaR1 and lipoxins were shown in human vagal tissue (71). While biochemical routes were proposed, evidence was not provided. As another example, the same PD isomers were shown in mouse thrombi using reverse-phase LC (72). The 17-epi-PD1 is also known as “aspirin-triggered-PD1”. As aspirin was not administered, it is not clear how it was formed enzymatically. Further complicating this, in papers reporting multiple isomers, calculated values often show that one or two epimers of each lipid dominate quantitatively. For example, in a study of murine inflammatory arthritis, 17*R*-RvD3, 7*S*,14*S*-diHDHA and 10*S*,17*S*-diHDHA (PDX) were more abundant than their SPM isomers RvD3, MaR1 and PD1 (73). As 17*R*-RvD3 is proposed to be generated by acetylated COX (74), it is again unclear where it originated from, as aspirin was not administered. Overall, complex tissues have often been reported to contain a mixture of many SPMs and their isomers, making conclusive assignment of their origin impossible. Notably, the above studies used methods that have now been shown not to adhere to community norms, and also included illustrations in place of raw data, making evaluation of the claims impossible.

To address the biosynthetic origin of SPMs in complex tissues, further confirmation is needed using disease models where one or more of the proposed biosynthetic enzymes are genetically deleted, combined with LC-MS/MS SPM analysis using chiral chromatography. While some early studies used chiral analysis to structurally annotate SPMs derived from purified or recombinant enzyme systems (75, 76), none appear to have applied this to the analysis of complex tissues or cells, meaning that the identity of lipids that co-elute with SPM standards was not clearly established. Generally, a stereochemically defined standard was compared with retention time and MS/MS spectra derived from reversed phase chromatography, and assignment of chirality inferred (77, 78, 79, 80). Aiming to address this issue, in 2014, a chiral method demonstrated the presence of multiple epimers of LXA_4_ in cell culture supernatants from stimulated granulocytes, and the co-incubations of granulocytes with platelets (81). Several other SPMs from DHA, including RvD1, 17-epi-RvD1, RvD2, MaR1 and PDX, and the EPA lipid RvE1 were absent. A second paper using chiral separation showed similarly how reverse-phase separation alone leads to false-positive reporting of LXA_4_ from cultured cells, if used without chiral confirmation (82). These studies raise significant questions as to the identity of lipids claimed to be present in early studies on SPMs, further calling into question their biochemical origin in complex tissues.

One recent study partially addressed this using 12/15-LOX (*Alox15*) knockout mice, which generate reduced amounts of precursor mono-hydroxy SPM substrates on challenge with skin wounding, including 17-HDHA (83). Only one SPM was putatively detected in wild-type wounds, RvD5. This was previously proposed to originate either from secondary oxygenation of 17*S*-HDHA by 5-LOX (*Alox5*) at C7, or alternatively through oxygenation of DHA at C7 by 5-LOX, followed by 12/15-LOX oxidation at C17, forming 7*S*,17*S*-diHDHA (49, 84). RvD5 is a diHDHA, meaning that exclusive non-enzymatic formation would result in four isomers which all co-elute in reversed phase-chromatography (50), although two diastereomers theoretically could be separated. Chiral analysis in wound tissues suggested several RvD5 isomers were present, like that seen in improperly stored human plasma (50).

### The use of ELISAs as an alternative method for SPM quantification

Aside from LC-MS/MS, another way to quantify oxylipins, including SPMs, is enzyme-linked immunosorbent assays (ELISA). Although these have been around for decades for PG and other oxylipins, these assays have long been recognized to have significant qualitative and quantitative problems. A recent study shows how ELISAs for SPMs, in particular RvD1 and RvD2, can detect signals in plasma or serum samples, which, according to LC-MS/MS, contain no detectable levels of the lipids (85). Furthermore, incorrect processing of plasma by incubation at 37 °C for extended periods increased the ELISA signals, despite no RvD1 and RvD2 being detected using LC-MS/MS. Manufacturers recommend that data obtained using these kits should be used as a guide and always need to be verified using LC-MS/MS. However, this is not generally being done, with many SPM studies being published using ELISA detection alone (57, 86, 87, 88, 89, 90, 91, 92, 93, 94, 95). In light of the above-mentioned aspects, claims made using this method (ELISA) without verification by LC-MS/MS need re-evaluation.

### Commercial assays for SPMs using LC/MS/MS

Aside from research laboratories, commercial services for SPM analysis using LC-MS/MS exist. Solutex, offers an LC-MS/MS service as described in several publications (96, 97, 98), with LOD based on S/N of >5:1 based on spiking in blanks, without mention of assessing SPM signals in matrix. It also describes matching 6 diagnostic ions in product ion spectra, like methods that do not adhere to community norms. Ambiotis runs an analytical LC-MS/MS service that includes SPMs (https://www.ambiotis.com/en/clinical-trials-spm-mass-spectrometry.html#list-of-mediators). Recent papers citing their method do not include LOD/LOQ criteria. Furthermore, papers using these methods show no chromatograms to evidence the presence of SPMs in samples (99, 100, 101). Last, Cayman provides a commercial assay that adheres to community-agreed approaches (16), but so far this has not reported detection of SPMs in any publications.

### Summary on SPM analytics

In summary, many studies on SPMs have applied LC-MS/MS methods that do not conform to expected standards. Combined with this, ELISAs continue to be used without LC-MS/MS verification, and ex vivo autoxidation has recently emerged as a potential significant source of the lipids and their isomers. These issues question many claims about the presence of endogenous SPMs, irrespective of whether their formation is in quantities sufficient to drive the resolution of inflammation.

## Enzymatic synthesis of SPMs in cells and tissues

The majority of SPMs are di- or trihydroxylated PUFA derivatives generated through the sequential activity of LOX enzymes, particularly 5-LOX (*ALOX5*) or 15-LOX (*ALOX15*), followed by a second activity, usually another LOX enzyme generating either a dihydro(pero)xide or an LTA_4_-related allylic epoxide that hydrolyzes to SPMs. In the case of the LTB_4_-related oxylipins MaR1 and PD1, the allylic epoxide is converted to a LTB_4_-related structure by a putative hydrolase that has not been identified. Participating LOX include human or murine platelet 12-LOX (*ALOX12*, *Alox12*), human 15-LOX1 (*ALOX15*), human 15-LOX2 (*ALOX15B*), or murine 12/15-LOX (*Alox15*). SPMs are also proposed to be formed by a 12- or 15-LOX acting independently (Fig. 1). Generally, monohydroxylated LOX-derived oxylipins are regarded as SPM precursors rather than SPMs themselves. Although LOXs produce abundant mono-hydroxy oxylipins from PUFAs, their capacity to biosynthesize most SPMs appears to be very limited. As discussed in greater detail below, SPM formation is reliably detected only with purified enzymes supplemented with substrates, following addition of high amounts of mono-hydroxy precursors to cells expressing LOX isoforms for secondary oxidation, or through addition of excess PUFAs such as AA or DHA to cells (84, 102, 103, 104, 105, 106, 107, 108, 109, 110, 111). Given the expression patterns of these LOX enzymes, SPM biosynthesis is largely confined to leukocytes and is frequently suggested to depend on transcellular transfer of a monohydroxy intermediate from one cell type, such as neutrophils, epithelial cells, or platelets, to another, such as macrophages or neutrophils (107, 112, 113, 114). Here, we consider the cellular and enzymatic generation of individual SPM families. Data are summarized from studies considered to have used reliable methods and/or have shown clearly discernible chromatographic peaks and that have been replicated by more than one laboratory, unless otherwise stated.

### Maresins

Maresin 1 (MaR1) and MaR2 were proposed as DHA derivatives of a 12-LOX (*ALOX12*) pathway in macrophages, with conversion of 14-HPDHA to a 13*S*,14*S*-epoxide intermediate and its further transformation to maresins by putative epoxide hydrolases (Fig. 1) (111). Later, it was shown, using recombinant human enzymes, that 15-LOX1 (*ALOX15*) could be involved (Fig. 1) (103). MaR1 has an LTB_4_-related structure (7*R*,14*S*-dihydroxy with an 8-*trans*,10-*trans*,12-*cis* conjugated triene), but it is not a product of LTA_4_ hydrolase that forms LTB_4_ (115). MaR2 is a 13*R*,14*S*-diHDHA that can be prepared by reaction of synthetic LTA_4_-type 13,14-epoxide with soluble epoxide hydrolase (111). Reliable formation of maresins has only been reported from recombinant enzymes or when intermediate precursors were added to leukocytes (103, 109, 110, 111). Beyond this, cellular formation of maresins from endogenous substrate is low, even after bacterial stimulation of M2-like human macrophages (47, 48, 49), despite formation of large amounts of the MaR precursor 14-H(p)DHA. Notably, 14-H(p)DHA levels are reported to be several orders of magnitude higher than maresin formation (for review, see Table 2 in (116)). We note that these studies used LC methods which, as outlined earlier, do not conform to community norms (7). Furthermore, in another publication claiming MaR2 formation, no chromatograms were shown, making data evaluation impossible (117). Overall, reliable studies indicate that there are at most trace levels (low pg amounts per million cells) of MaRs generated in macrophages, even in the presence of large amounts of their fatty acyl or mono-hydroxy precursors, while studies in tissues used methods where data cannot be verified.

### Protectins

Analogous to the proposed formation of maresins from 13*S*,14*S*-epoxy-DHA, an 16*S*,17*S*-epoxy-DHA intermediate formed by 15-LOX has been proposed to yield the protectins, PD1 (also named NPD1, Neuroprotectin D1) and PD2 (118). The formation of protectins was proposed from studies where cells or neuronal tissues were supplemented with exogenous DHA or oxylipin precursors (67, 118, 119) or studies where recombinant human 12-LOX, 15-LOX1 or 15-LOX2 was supplied with DHA substrate (104). PD1 was originally prepared and assigned an LTB_4_-related structure (a *trans-trans-cis* conjugated triene) by biosynthesis using soybean 15-LOX-1 (120). The structure was deduced based on UV and LC-MS analyses both as a product or by-product of soybean LOX activity (albeit going against all precedent and not independently confirmed) (120), and reported as a product of biosynthesis in mammalian cells (121, 122). As noted early on, it is well-precedented that soybean LOX will produce a 10*S*,17*S*-diol with 11-*trans*,13-*cis*,15-*trans* conjugated triene and not the a *trans-trans-cis* conjugated triene assigned to PD1 (66, 123). If isomerization of the well-established soybean LOX product occurred, it would likely give an all-*trans* conjugated triene, but formation of the PD1 structure, a 10*R*,17*S*-diol with *trans, trans,cis* bonds, has never been demonstrated or independently established. PD1 was prepared by total chemical synthesis by four independent groups, and the compound is available commercially (124, 125, 126, 127). Over the past decade, the existence of PD1 as a cellular product has been challenged based on its similarity to other 10,17-dihydroxy docosanoids (50, 123, 128, 129). The LTB_4_-related structure would not be expected as a significant product, based on known mechanisms of polyunsaturated autoxidation (130), as recently confirmed (131), and thorough LC-MS/MS analyses established that PD1 is not among the DHA-related isomers formed in non-enzymatic transformations (50). PD1 can be distinguished from related dihydroxy isomers based on a careful comparison of UV spectra (128), differences in the ratios of product ions by monitoring different transitions (16, 129), and separated from others by normal-phase HPLC (128), although the latter was not applied in the original misidentification of the product from soybean LOX-1 activity (120). Other investigations of 12/15-LOX metabolism of DHA in murine peritoneal macrophages failed to detect PD1 (128). In sum, PD1 has not been demonstrated to form enzymatically, and there is no compelling evidence for its in vivo formation.

Another protectin, PDX, with altered double bond geometry is formed by double lipoxygenation of DHA at C10 and C17 (68). Furthermore, only very low amounts of PDs have been observed in M2-like macrophages (47, 48, 49), although as stated earlier, these three studies used methods that do not follow community norms. Importantly, the precursor 17-H(p)DHA was detected, exceeding reported PD levels by several orders of magnitude (for review, see Table 2 in (116)).

### Lipoxins and resolvins

The biosynthesis of lipoxins from AA or EPA, or D and E series resolvins from EPA and DHA, has been proposed to involve two consecutive oxygenase reactions catalyzed by 5-LOX either before or after oxygenation by a 12- or 15-LOX isoform (e.g. 12-LOX, 12/15-LOX, 15-LOX1 or 15-LOX2) (Fig. 1) (1, 2, 63). The sequence of oxygenation, specifically whether 5-LOX oxygenates first or second depends on the cell types and experimental conditions (for further reading see (6)). Both are considered in more detail below.

#### The 5-LOX:12/15-LOX pathway

Here, oxygenation is proposed to be first mediated by 5-LOX, with the initial products from AA, EPA or DHA being 5*S*-HpETE, 5*S*-HpEPE or 7*S*-HpDHA, respectively. These oxylipins can then serve as substrates for 12- or 15-LOX, resulting in a series of dihydroxylated products that notably include 5*S*,15*S*-diHETE and 7*S*,17*S*-diHDHA (RvD5), both consistently detected in human M2 macrophages (116, 132, 133) (Fig. 1). Studies with purified enzymes suggest that 15-LOX2 might be involved in RvD5 formation (105).

In addition to the monohydroxylated oxylipins, 5-LOX produces the unstable allylic epoxide LTA_4_ from AA (134). This has been proposed to be required for the formation of trihydroxylated SPMs such as lipoxins, since it can serve as substrate for secondary oxygenation by a 12-LOX or 15-LOX isoform (e.g. 12-LOX, 12/15-LOX, 15-LOX1 or 15-LOX2) in leukocyte/platelet coincubations (107, 112, 113). However, epoxide formation by 5-LOX using DHA as a substrate has not been demonstrated (135) so trihydroxylated resolvins of the D series cannot be initially formed via the 5-LOX:12/15-LOX pathway. Due to this, formation of RvD1 and RvD2 requires addition of the epoxide intermediate to neutrophils and macrophages (136). Although prominent lipoxin formation has been observed in neutrophil/platelet coincubations where neutrophil-derived LTA_4_ is converted by platelets to lipoxins (107, 112, 113), formation of lipoxins in blood is low or undetectable even after stimulation (137, 138). Here, LTA_4_ may be bound to albumin and not available for conversion by platelet 12-LOX (139). This is supported by data showing that plasma proteins drastically slow down the further metabolism of LTA_4_ released by neutrophils (140). Similarly, LXA_5_, 15-epi-LXA_5_ and RvE4 were recently detected after addition of excess racemic 15-HEPE to human neutrophils (44).

Several publications claim that 5-LOX is involved in the formation of the D-series resolvins RvD3, RvD4, and RvD6 (141, 142). In this context, 5-LOX is hypothesized to first form 4*S*-HDHA from DHA. Following this, the trihydroxylated D-series resolvins RvD3, RvD4, and RvD6 are postulated to form via secondary oxygenation of 4*S*-HDHA by a 12-LOX or 15-LOX isoform. However, no published experimental evidence exists to support this claim. Although 4-HDHA formation by 5-LOX has been demonstrated in murine granulocytes (143), human granulocytes only form small amounts of this lipid after DHA supplementation (135), while another study demonstrated that recombinant human 5-LOX is unable to generate 4*S*-HDHA from DHA (105). Notably, 4-HDHA and 20-HDHA are the two most common autooxidation products of DHA (144, 145). This strongly suggests that 4-HDHA may be formed in cell incubations non-enzymatically. In line with this, the formation of 4-HDHA is not inhibited by 5-LOX and FLAP inhibitors in stimulated human M1 and M2 macrophages (146), while 4*S*-HDHA-derived D-series resolvins are barely detected in human leukocytes (116). Of note, RvD3 and RvD4 were recently demonstrated in human neutrophils, but only when incubated with the synthetic precursor oxylipin 4*S*,5*S*-epoxy-17*S*-HDHA (147). However, considering that a synthetic precursor was added, this study represents nonphysiological conditions and does not address whether and how these mediators might be formed in leukocytes in vivo and if so, which enzymes are involved. In summary, there is no evidence that 5-LOX:12/15-LOX acting in tandem can form trihydroxylated resolvins in vivo.

#### The 12/15-LOX:5-LOX pathway

The 12/15-LOX:5-LOX pathway is often referred to as a primary route for SPM formation. Here, AA, EPA and DHA are first oxygenated by human 15-LOX1 (ALOX15) or 15-LOX2 (ALOX15 B) or CYPs to form the monohydro(pero)xy-fatty acid intermediates: 15-H(p)ETE, 15-H(p)EPE, 18-H(p)EPE or 17-H(p)DHA. Following this, these mediators are further metabolized by 5-LOX to form dihydroxylated and trihydroxylated SPMs, such as lipoxins and resolvins. For the formation of trihydroxylated SPMs (Table 1) via this pathway, the generation of an epoxide at carbons 5 and 6 (with AA and EPA-derived oxylipins) and at carbons 7 and 8 of 17-H(p)DHA by 5-LOX is proposed to be required. However, current evidence indicates that the monohydro(pero)xylated oxylipins are poor substrates for purified 5-LOX (for review, see Table 1 in (116)) and FLAP is also required for their acceptance as substrates in cells (see also below). Of note, formation of dihydroxylated SPMs such as 5*S*,15*S*-diHETE, RvD5 and RvE4 is 1–2 orders of magnitude lower than that of their monohydroxylated precursors, even in stimulated M2-like macrophages which prominently express FLAP, 5-LOX and 15-LOX1 (47, 48, 108, 116, 146, 148, 149, 150). In contrast to the monohydroxylated and dihydroxylated oxylipins, formation of trihydroxylated SPMs in leukocytes is extremely low or undetectable by LC-MS/MS. This is likely due to extremely inefficient conversion of oxylipin precursors to the corresponding epoxides by 5-LOX via its LTA_4_ synthase activity, which up to now has been shown to require exogenous addition of 15-HETE or 17-HDHA (44, 151) (Fig. 1).

The biosynthesis of several E-series resolvins (RvE1, RvE2, RvE3) has been proposed to occur from 18*R*-HEPE (152, 153) (Fig. 1). However, the biosynthetic origin of this oxylipin in human cells is unclear since human LOX isoforms are unable to effectively generate 18*R*-HEPE from EPA (for review see (6)). An alternative source may be CYP enzymes, which can catalyze 18*R*-HETE formation from AA (154), although it is unknown whether analogous reactions occur from EPA. To the best of our knowledge, there is no report showing relevant formation of 18*R*-HEPE from EPA by human CYPs.

#### FLAP dependency

5-LOX-activating protein (FLAP) has long been known to be required for generation of 5-HETE and LTA_4_ from AA by 5-LOX in intact cells (155). Similarly, SPMs such as lipoxins generated via 5-LOX (acting via the 12/15-LOX:5-LOX route), are also considered to be FLAP-dependent (84, 107). Supporting this idea, FLAP is required for 5-LOX-dependent oxygenation of oxylipins including 12- or 15-HETE (156, 157). Alongside this, formation of trihydroxylated SPMs, such as LXA_4_ (Table 1) via the 5-LOX:12/15-LOX route is also considered FLAP-dependent. For example, cellular formation of the precursor LTA_4_ requires FLAP, while RvD5 biosynthesis via the 12/15-LOX:5-LOX route was recently confirmed to be FLAP-dependent (84). However, contradicting this, RvD5 formation in human M2-like macrophages via the 5-LOX:12/15-LOX pathway was claimed to be FLAP independent in three studies (49, 146, 158). Strikingly, it was also claimed that inhibition of FLAP strongly upregulated SPM biosynthesis. Oddly, in two of these studies, the FLAP inhibitor MK886 reduced formation of the precursor 7-HDHA in M1 cells while simultaneously increasing its formation in M2 cells (49, 146). Several studies have demonstrated that 5-LOX and FLAP are more highly expressed in human M1 cells, so the observation that MK886 prevents 7-HDHA formation in these cells is fully in line with its predicted role in supporting 5-LOX activity (155). How FLAP inhibitors might instead promote activity of 5-LOX to oxidize DHA in one cell type but not another is unclear, also considering that MK886 blocks 7-HDHA formation in human neutrophils (159). FLAP-independent RvD5 formation has only been observed when M2-like cells were stimulated with Staphylococcus aureus-conditioned medium but not with calcium ionophore (146) suggesting that this effect is stimulus and cell-type -dependent. Where SPM generation is FLAP independent, it is possible that 5-LOX is not involved and a role for autoxidation driven by macrophage ROS from sources that include NADPH oxidase and myeloperoxidase, can instead be considered (160). This is supported by the observation that the direct 5-LOX inhibitor zileuton was much less effective at blocking formation of 5-HETE generation in human M2-like macrophages than in M1 (146). This suggests significant 5-LOX independent generation of the respective oxylipins in M2 cells under these conditions. Chiral LC-MS/MS analysis would be required to elucidate the stereochemistry of the formed oxylipins to gain more insights into their origin. Furthermore, it is noted that studies proposing FLAP independent SPM formation have all used analytical methods that do not conform to community norms (49, 146, 158). Overall, studies suggesting a role for FLAP in elevating SPM formation in macrophages appear at odds with the proposed role of 5-LOX in this process and need to be re-evaluated.

A second area of discussion relating to the role of FLAP concerns the idea that cellular location of 5-LOX dictates whether SPMs or leukotrienes are formed in leukocytes, with cytosolic localization favoring SPM formation and translocation to the nuclear membrane fostering pro-inflammatory leukotriene formation (161). This assumption is based on the observation that inhibition of p38 MAPK reduces nuclear localization of 5-LOX and slightly reduces LTB_4_ formation whereas LXA_4_ formation was increased by 2-fold (161). However, contradicting this idea, the formation of lipoxins is completely FLAP-dependent (84, 107) and FLAP is located in the nuclear membrane (162). This questions the idea that SPM formation is spatially controlled, suggesting that based on current evidence, SPMs (if enzymatically formed) in human leukocytes would occur at the nuclear membrane, like leukotrienes.

### Aspirin-triggered SPMs

Another family of SPMs are termed “aspirin-triggered” (AT-SPMs), since they form through the action of aspirin-acetylated COX-2 (114, 163, 164). Here, monohydroxylated oxylipins with altered stereochemistry are formed which are proposed to then be converted to corresponding AT-SPMs. At present, the relevance of these lipids to the pharmacological actions of aspirin in vivo is entirely speculative. No evidence exists for their contribution to either the anti-inflammatory or anti-thrombotic actions of aspirin in vivo. Additionally, several studies reported detection of AT-SPMs in tissues where aspirin had not been administered, raising concerns about their artefactual non-enzymatic generation (71, 72, 73, 165).

### Summary of evidence on enzymatic and cellular generation of SPMs

The proposed biosynthetic routes for most SPMs require 5-LOX together with either 12/15-LOX (*Alox15*), 15-LOX1 (*ALOX15*), 15-LOX2 (*ALOX15B*), or 12-LOX (*ALOX12*, *Alox12*). Because 5-LOX is expressed almost exclusively in leukocytes, these cells are regarded as central contributors to SPM production. Although both M1-like and M2-like human macrophages express the full enzymatic machinery needed for SPM biosynthesis, their capacity to produce most 5-LOX-dependent dihydroxylated SPMs is limited, whereas trihydroxylated SPMs are barely detectable or not detectable at all. Similar limitations apply to MaRs and PDs. This is thought to reflect intrinsic constraints in LOX-mediated biosynthesis of the fatty acid epoxide intermediates and/or limited mobilization of endogenous fatty acid precursors.

Using methods adhering to current community guidelines (16), the lack of formation of most proposed SPMs in human macrophages and mouse tissues has recently been independently confirmed by Werz *et al.* (52). Interestingly, the authors interpreted the detection of trace amounts of a small number of dihydroxylated products, including RvD5, RvE4, PDX and LXA_4_ as support for the biological relevance of SPMs and for the hypothesis that these molecules act as endogenous mediators of inflammation resolution. However, the overall dataset supports a different conclusion. Despite the presence of the complete enzymatic machinery required for SPM biosynthesis, the vast majority of proposed SPMs remained undetectable, while those detected were formed only in minute amounts. The proposed endogenous role of those SPMs would require signaling by high-affinity receptors. Originally, a number of G-protein-coupled receptors were proposed for several SPMs (152, 166, 167, 168, 169, 170, 171, 172), but most of these assignments could not be confirmed by independent labs (6, 169, 173, 174, 175, 176, 177, 178, 179, 180, 181, 182). The issue of potential SPM receptors is therefore controversial, and it remains unclear whether specific high-affinity G-protein coupled receptors for SPMs exist (6). Other mechanisms that could mediate effects of high pharmacological doses of protectins, maresins, and D-series resolvins have been recently published. These include their action as allosteric modulators of the EP4 receptor, low-affinity activators of PPARγ, or stimulation of Nrf2 by SPM metabolites (173, 180, 183, 184, 185, 186). In the case of EP4, it was shown that DHA-derived SPMs can act as positive allosteric modulators of the receptor through interaction with an intracellular binding site, leading to an increase in PGE_2_-induced cAMP formation and PI-3-kinase activity thereby promoting anti-inflammatory and pro-phagocytic effects (173).

Taken together, the available biosynthetic data make it highly unlikely that immune cells generate most proposed SPMs (Table 1) from endogenous fatty acid precursors in amounts sufficient to function as physiologically relevant mediators of inflammation resolution.

## Summary and Outlook

SPMs have been proposed to function as endogenously produced lipid mediators that coordinate the resolution of inflammation. However, the evidence reviewed here suggests that several core elements of the SPM paradigm require major reassessment, and their role as endogenous drivers of inflammation resolution remains unproven. The original concept is based on quantitative methods with fundamental limitations, possible artefactual generation through ex vivo autoxidation, and incorrect assignment as GPCR ligands. Future investigations into SPM biosynthesis and physiology should follow community-recommended analytical standards (16) and apply the strict IUPHAR criteria for receptor identification and validation (187).

## Conflict of interest

The authors declare that they have no conflicts of interest with the contents of this article.
